# Draft Genome Sequence of a Necrotoxigenic *Escherichia coli* Isolate

**DOI:** 10.1128/genomeA.01152-15

**Published:** 2015-10-01

**Authors:** Sadjia Bekal, Alex Lin, André Vincent, Chrystal Berry, Matthew Gilmour, Éric Fournier, Jean-Charles Côté, Cécile Tremblay

**Affiliations:** a Laboratoire de santé publique du Québec, Sainte-Anne-de-Bellevue, Québec, Canada; b Département de Microbiologie, infectiologie et immunologie, Université de Montréal, Montréal, Québec, Canada; c Centre Hospitalier Affilié Universitaire, Hôtel-Dieu de Lévis, Lévis, Québec, Canada; d Public Health Agency of Canada, National Microbiology Laboratory, Winnipeg, Manitoba, Canada

## Abstract

Here, we present the draft genome sequence of a necrotoxigenic *Escherichia coli* strain isolated from a patient following a very rapidly evolving, lethal necrotizing fasciitis.

## GENOME ANNOUNCEMENT

*Escherichia coli* is a bacterial commensal of the intestinal tract of warm-blooded animals, including humans. It is Gram-negative, facultative anaerobe, and rod-shaped (1). Although most *E. coli* strains are harmless, several others are the etiological agents of various diseases. The latter can be divided into intestinal (diarrheagenic) and extraintestinal pathogenic *E. coli* (ExPEC) strains (2, 3). The intestinal pathogenic group comprises at least six pathotypes: enteropathogenic (EPEC), Shiga-toxin producing enterohemorrhagic (STEC/EHEC), enterotoxigenic (ETEC), enteroaggregative (EAEC), enteroinvasive (EIEC), and diffusely adherent *E. coli* (DAEC). The ExPEC comprises at least three named pathotypes: uropathogenic (UPEC), newborn meningitis-causing *E. coli* (MENEC), an unnamed pathotype which encompasses the strains that cause septicemia in humans and animals, and necrotoxigenic *E. coli* (NTEC) (2, 3). A characteristic cytotoxic necrotizing factor (Cnf1 or Cnf2) is synthesized by uropathogenic and necrotoxigenic *E. coli* strains (4, 5).

Necrotizing fasciitis, commonly referred to as “flesh-eating disease” is a rare but severe disease characterized by the necrosis of the subcutaneous tissues and fascia (6, 7). It is usually caused by *Streptococcus pyogenes* (8, 9), sometimes *Staphylococcus aureus* (10) or by a mixture of microorganisms including *Streptococcus*, *S. aureus*, *Enterobacteriaceae*, and some anaerobes (11, 12). On rare occasions, necrotoxigenic *E. coli* was identified as the etiologic agent in chronically ill patients (13–20) or infants following surgery (21, 22). Recently, we reported a lethal case of very rapidly evolving necrotizing fasciitis. The bacterial isolate was identified as *E. coli* and designated LSPQ A134697 (Laboratoire de Santé Publique du Québec, strain A134697). DNA microarray revealed the presence of several toxin genes, including the cytotoxic necrotizing factor 1 gene *cnf1* (23).

We present here the draft genome sequence of LSPQ A134697. A sequencing library was prepared using the Nextera XT library preparation kit (Illumina, Inc., San Diego, CA, United States). Sequencing was performed on an Illumina MiSeq platform with the MiSeq Reagent Kit v2, 500 cycles to achieve 83× average genome coverage. The quality of the raw sequence data was checked using FastQC (http://www.bioinformatics.babraham.ac.uk/projects/fastqc/). The sequence reads were *de novo* assembled into contigs using SPAdes v3.5 (http://bioinf.spbau.ru/spades [24]). A total of 81 contigs, ranging in size from 505 bp to 621,560 bp, with a *N*_50_ of 244,924 bp, for a total length of 5,228,778 bp with an average G+C content of 50.6% was generated. They were annotated using the NCBI Prokaryotic Genome Automatic Annotation Pipeline v2.10 (PGAAP) (25). A total of 5,126 protein-coding sequences, 81 tRNAs, 17 rRNAs (5S, 16S, and 23S), and 81 pseudogenes are predicted. The *cnf1* gene is located on contig 24, at positions 42,141 to 45,185. Further analysis of this genome and comparison with others will be presented elsewhere.

This is the first annotated draft genome sequence of a necrotoxigenic *E. coli* isolate.

### Nucleotide sequence accession number.

The annotated genome sequence was deposited in GenBank under accession no. LELX00000000.
